# Phase I, dose-escalation, clinical trial of MVA-Brachyury-TRICOM vaccine demonstrating safety and brachyury-specific T cell responses

**DOI:** 10.1186/2051-1426-3-S2-P132

**Published:** 2015-11-04

**Authors:** Christopher R Heery, Renee Donahue, Lauren Lepone, Italia Grenga, Jacob Richards, Simon Metenou, Romaine I Fernando, Ulrike Dirmeier, Harpreet Singh, Ravi Madan, James L Gulley, Jeffrey Schlom

**Affiliations:** 1 Laboratory of Tumor Immunology and Biology, Center for Cancer Research, National Cancer Institute, National Institutes of Health, Bethesda, MD, USA; 2 Bavarian Nordic GmbH, Martinsried, Germany; 3 Genitourinary Malignancies Branch, Center for Cancer Research, National Cancer Institute, National Institutes of Health, Bethesda, MD, USA

## Background

Brachyury is a tumor-associated antigen and transcription factor that drives the epithelial-to-mesenchymal transition in human carcinomas. This Phase I study assessed whether patients with advanced cancer or chordoma, a rare tumor of the notochord that overexpresses brachyury, can elicit brachyury-specific T cell responses following vaccination with MVA-Brachyury-TRICOM.

## Methods

Patients with advanced cancer (n=25) or chordoma (n=13) enrolled on a Phase I clinical trial of MVA-brachyury vaccine (NCT02179515). Dose escalation was performed following 3+3 design in 3 dose levels (DL1=2x10^8^, DL2=4x10^8^, DL3=8x10^8^ plaque forming units, with vaccine administered in 3 cycles every 4 weeks). After safety was established, expansion cohorts were enrolled at DL2 and DL3 to compare brachyury-specific-T cell responses. Peripheral blood mononuclear cells from pre- and post-vaccination (~day 29, 57, 85, and 176) were stimulated with brachyury or HLA (negative control) 15-mer peptide pools and analyzed for brachyury-specific-T cell responses by intracellular staining of CD4 and CD8 T-lymphocytes for the cytokines IFN-γ, TNF, and IL-2, and the degranulation marker CD107a.

## Results

In total, 3 patients enrolled on DL1, 17 on DL2, and 18 on DL3. On DL2 and DL3, 1 and 2 patients, respectively, were not evaluable for safety or immune responses and replaced. MVA-brachyury was well tolerated with no dose limiting toxicities. The maximal tolerated dose was not reached. Two deaths occurred on study, both due to complications of rapid disease progression, unrelated to vaccine. Two other serious adverse events (AEs) occurred, a hip fracture after a fall and a colonic obstruction due to disease progression. No serious adverse event was related to vaccine. AEs occurring in >2 unique patients included diarrhea (7.9%), fever (18%), flu-like symptoms (34%), and injection site reaction (74%). One grade 3 adverse event, diarrhea, was related to vaccine, and resolved without intervention after 48 hours. All other adverse events related to vaccine were grade 1 or 2 with short duration. Immune responses were analyzed in 29 patients. Brachyury-specific T cell responses were observed at each dose level: 66% (2/3) of patients at DL1, 80% (12/15) at DL2, and 90% (10/11) at DL3. At DL2 and DL3, ~80% of the patients that developed brachyury-specific-T cells demonstrated responses in both CD4 and CD8 T-lymphocytes.

## Conclusions

These findings show for the first time that advanced cancer patients can be safely immunized with an MVA-based vaccine targeting brachyury, and can develop brachyury-specific T cell immune responses. These results warrant further studies using this vaccine in additional cohorts of cancer patients.

